# Increased Protein with Decreased Carbohydrate Intake Reduces Postprandial Blood Glucose Levels in Women with Gestational Diabetes: The iPRO Study

**DOI:** 10.1089/whr.2022.0012

**Published:** 2022-08-31

**Authors:** Kimberly K. Trout, Charlene W. Compher, Cara Dolin, Carrie Burns, Ryan Quinn, Celeste Durnwald

**Affiliations:** ^1^ Department of Family and Community Health, University of Pennsylvania, Philadelphia, Pennsylvania, USA.; ^2^ Department of Obstetrics and Gynecology, University of Pennsylvania, Philadelphia, Pennsylvania, USA.; ^3^ Departments of Biobehavioral Health Sciences, and University of Pennsylvania, Philadelphia, Pennsylvania, USA.; ^4^ Department of Medicine, University of Pennsylvania, Philadelphia, Pennsylvania, USA.

**Keywords:** midwifery, gestational diabetes, macronutrients, protein

## Abstract

**Introduction:**

There is an urgent need to establish an evidence base for recommendations regarding proportions of macronutrients for optimized nutritional management of gestational diabetes mellitus (GDM). Our study compared isocaloric diets in women with GDM that differed in protein and carbohydrate content with fats held constant. We hypothesized that the glucose area under the curve (AUC) would be lower with the higher protein/lower carbohydrate diet.

**Research Design and Methods:**

This study used a random order crossover design within a controlled research unit environment. Nineteen women were randomized to treatment, with 12 participants completing both arms of the study. Blood sampling occurred preprandially and at *t* = 30, 60, 120, and 180" relative to meals. Inclusion criteria were confirmed diet-controlled GDMA1, singleton gestation, and with no pre-existing medical comorbidities. Mean gestational age at entrance to study = 32 (±1.76) weeks. Mean prepregnant body mass index of participants = 28.7 (±5.3) kg/m^2^ Participants were randomly assigned initially to either an increased protein/low carbohydrate (iPRO30%/CHO35%) diet or a lower protein/higher carbohydrate (LPRO15%/CHO50%) diet for a 36 hour inpatient stay on the research unit. All meals and snacks were prepared in a metabolic kitchen. After a 3–7 day washout period, participants were randomized to the opposite treatment.

**Results:**

On day 2 (with confirmed overnight fasting), the average 3-hour pre- through postprandial glucose AUC was lower in iPRO30%/CHO35% treatment arm (17395.20 ± 2493.47 vs. 19172.47 ± 3484.31, *p* = 0.01).

**Conclusion:**

This study is the first to demonstrate that a higher protein, lower carbohydrate meal, especially at breakfast, can result in lower postprandial blood glucose values in women with gestational diabetes. A lack of statistically significant differences at other collection time points could have been due to several factors, but most likely due to small sample size. Longer term outcomes of a higher protein diet, including maternal glycemic control, nitrogen balance, and impact on fetal growth outcomes, are needed.

## Introduction

Gestational diabetes mellitus (GDM) impacts women and their infants, with increasing evidence supporting the concept that the intrauterine environment in a pregnancy affected by GDM may have a lifelong impact on both mother and child.^1^ As one of the most common pregnancy complications, GDM affects up to 6%–7% of pregnancies in the United States, with higher prevalence in racial/ethnic groups at greater risk for health disparities (Black, Latina, and Asian women).^1^

Since control of dietary energy intake is a key feature of GDM management, there is an urgent need to establish an evidence base for recommendations regarding proportions of macronutrients for optimized nutritional management of the condition. Carbohydrate restriction is considered the primary diet therapy for GDM, yet, evidence is scant regarding the optimal amounts of carbohydrate, protein, and fat for blood glucose control in patients with GDM. A systematic review and meta-analysis of randomized controlled trials that involved women with GDM examined the effects of modified dietary interventions on maternal glucose control and infant birth weight.^2^ The conclusion of the review was that dietary interventions resulted in lower mean glucose levels when compared with control participants who did not experience dietary interventions, but the analysis was not able to discern one type of dietary advice as superior to another.

The most recent Cochrane review concluded that there is insufficient evidence to make recommendations for dietary treatment of GDM.^3^ The review examined 10 different types of specific dietary advice.

The comparisons included (1) a low-moderate glycemic index (GI) diet compared with a moderate-high GI diet (four trials)^4–7^; (2) an energy-restricted diet compared with no energy restriction (three trials)^8–10^; (3) a “Dietary Approaches to Stop Hypertension (DASH)” diet rich in fruits, vegetables, whole grains, and low-fat dairy products compared with a control diet (three trials)^11–13^; (4) a low-carbohydrate diet compared with a high-carbohydrate diet (two trials)^14,15^; (5) a high unsaturated-fat diet with a low unsaturated-fat diet (two trials)^16,17^; (6) a low-GI diet with a high-fiber moderate-GI diet (one trial)^18^; (7) dietary recommendations with concomitant diet-related behavioral advice when compared with dietary recommendations alone (one trial)^19^; (8) a soy protein-enriched diet compared with no soy protein (one trial)^20^; (9) a high-fiber diet with a standard-fiber diet (one trial);^21^ and (10) an ethnic-specific diet with a standard healthy diet (one trial).^22^

The review found no clear differences between the different types of dietary advice on multiple parameters, including the number of women with hypertensive disorders of pregnancy, large-for-gestational age infants, perinatal deaths, the development of type 2 diabetes in the mother and a composite outcome of neonatal deaths or ill-health of the infants.^3^ There was a reduction in the incidence of cesarean birth with the DASH diet (relative risk = 0.53 [95% confidence interval 0.37–0.76]).^11,13^ None of the included trials reported on later outcomes during childhood for the infants.^3^

The American Diabetes Association (ADA) had recommended in 1995 that women with GDM restrict carbohydrates to 35%–40% of total caloric intake, but retracted this recommendation in 2004, citing insufficient or poor quality evidence. In 2021, the ADA maintains that there is insufficient data to offer precise dietary advice for women with GDM and acknowledge that there is increasing evidence supporting the notion that dietary advice is not “one size fits all” and that dietary therapy should be tailored to the individual.^23^

Previous studies have manipulated levels of carbohydrate, adjusting fat intake to compensate, but we have found none in women with GDM that have evaluated the impact of varied protein loads with compensatory carbohydrate manipulation on blood glucose control in a controlled feeding environment. In patients with type 2 diabetes (a form of diabetes similar to GDM in that both are characterized by insulin resistance), increased protein, especially whey protein, may have a beneficial effect not only on glucose and insulin metabolism, but also on serum lipids, lipoproteins, vascular function, and blood pressure.^24^ There is evidence that proportions of macronutrients can have an impact on fetal growth patterns, yet, beyond Institute of Medicine guidelines setting a minimal intake of carbohydrates at 175 g/day and minimal intake of protein at 71 g/day (or 1.1 kg/day), evidence is conflicting regarding what is optimal.^25–27^

This study is not designed to address long-term outcomes of varying macronutrient intake on fetal growth. The purpose of this study was to investigate the impact of a higher protein and lower carbohydrate diet on maternal blood glucose levels in a well-controlled feeding trial in women with GDM not treated by insulin or other medication.

The primary aim was thus to evaluate glucose and insulin responses to meals where energy was provided as 30% protein, 35% carbohydrate, and 35% fat (iPRO30%/CHO35%) or 15% protein, 50% carbohydrate, and 35% fat (LPRO15%/CHO50%) using a random order crossover study design. We hypothesized that the higher protein and lower carbohydrate diet would result in lower mean premeal through postprandial 3-hour glucose area under the curve (AUC) and lower mean premeal through postprandial 3-hour insulin AUC (as protein is not expected to have as much of an immediate impact on insulin secretion as carbohydrates). Exploratory aims were evaluation of free fatty acids (FFA), cortisol, and proinflammatory cytokines, as these biomarkers have been implicated in the development of insulin resistance.

## Methods

### Patient and public involvement

Participants were recruited from the University of Pennsylvania Health System (UPHS) obstetrical and midwifery practices in the City of Philadelphia via study flyers posted in waiting areas. The development of the study question was informed by patient expression of exasperation in controlling blood glucose levels. Patients were not involved in the design of the study. Inclusion criteria were diagnosis of GDM between 28 and 35 weeks gestation according to the 100 g, 3-hour glucose tolerance test (Carpenter and Coustan criteria).^23^

**FIG. 1.**
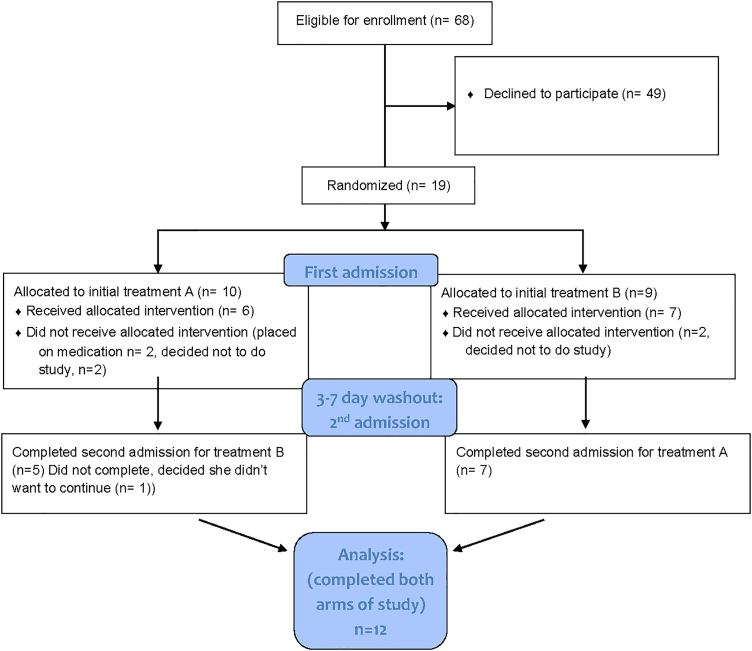
Study flow diagram.

An additional criterion was good dietary control of blood glucose (>80% of self-monitored blood glucose values meeting expected targets of <90 mg/dL fasting and <120 mg/dL 2-hour postprandial) over a minimum of 3 days to confirm diet-controlled GDMA1. Exclusion criteria were diabetes pre-existing the pregnancy, GDM requiring medication (insulin or oral agents), multifetal pregnancy, chronic hypertension or other serious medical disorders that would impact pregnancy, or inability to schedule two overnight admissions to complete the study requirements (Fig. 1).

### Study protocol

Study participants completed two 36-hour admissions to the Center for Human Phenomic Science (CHPS) unit of the Hospital of the University of Pennsylvania. Consented participants were randomly allocated (via sealed envelope) to the order of dietary treatment during the admission, with diet arms separated by a 3–7 day washout period at home. During the washout period, participants were instructed to follow the usual dietary advice prescribed by their health care provider and/or Certified Diabetes Care & Education Specialist.

The CHPS unit houses a metabolic kitchen managed by skilled research dietitians. Meals and snacks were provided to participants with total energy intake targeted to their individual requirements and food preferences during pregnancy but tailored to provide energy as either 30% protein, 35% carbohydrate, and 35% fat (iPRO30%/CHO35%) or 15% protein, 50% carbohydrate, and 35% fat (LPRO15%/CHO50%) as indicated by the treatment arm. In both diets, the fat intake was unchanged and at a level presumed to be sufficient to meet fatty acid requirements.^27^ The 15% protein intake meets the IOM recommendation of 1.1 g/kg/day, and 30% protein intake is not unusual during periods of controlled carbohydrate intake. Snacks were offered in between lunch and dinner (3 pm) and between dinner and bedtime (8 pm) and consisted of milkshakes that were either milk/fruit combination shakes or whey protein/fruit flavored shakes (UNJURY, Inc.: Sterling, VA).

Participants were blinded as to which treatment arm they were exposed to for each admission. Participants selected food choices from a menu that included several choices for each meal. These items could be tailored to specific food preferences or personal dietary restrictions (*e.g.*, no pork) and still maintain required macronutrient proportions for each of the treatment arms, as indicated. Menus were based on a minimum of 2000 kcal/day diet, ensuring that minimum Recommended Daily Allowance for carbohydrates (175 g/day) and protein (1.1 g/kg/day) would be achieved in both arms of the study. Calorie calculations were based on the Mifflin-St. Jeor formula, with an added 450 calories/day as recommended for the third trimester of pregnancy.

**FIG. 2.**
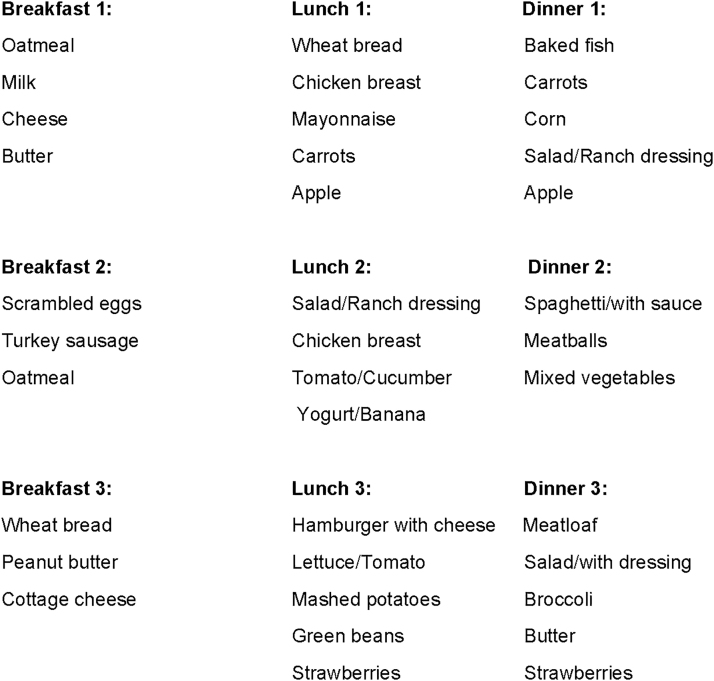
Sample menus. The same menus were used for both treatment arms and dieticians would vary portion sizes of items chosen by participants based on assigned treatment arm (*e.g.*, increased portion size of eggs for breakfast with iPRO30%/CHO35%).

Participants were given menus before admission with a variety of meal and snack choices that emphasized low to moderate GI foods (Refer to Fig. 2 ). Participants were instructed to circle foods on the menu that they wanted to eat and they were also instructed to cross out any foods that they did not want to eat. Simple sugars and fruit juices were not included in either diet arm. Protein and fat were obtained from both animal (meat, fish, dairy) and plant (peanut butter, beans) sources. Actual weighed food intake was entered into the Nutrition Data Systems for Research database (University of Minnesota) to confirm intake of energy for each meal and snack, as well as the daily total.

Data were evaluated by % kcal as protein, carbohydrate, and fat, with 20% kcal at breakfast, 25% kcal at lunch, 35% kcal at dinner, and 10% kcal for each of the two snacks.

Participants arrived at the CHPS unit between 6 and 7 am after an 8-hour overnight fast. Blood samples were taken at baseline (immediately before first bite of meal) and 30, 60, 120, and 180 minutes relative to each meal with the exception of the dinner meal on day 2, when postprandial samples were only obtained up to 60 minutes postdinner (to allow participants to be discharged to home and ease participant burden). After centrifugation at 3000 RPM at 4°C for 15 minutes, plasma samples were aliquoted and stored immediately in −80°C freezers until analysis. Blood samples were analyzed for glucose using a YSI 2900 analyzer (YSI: Yellow Springs Instruments, OH). Insulin, cortisol and cytokine levels (TNF-α, IL-6 and CRP) were measured via radioimmunoassay kits (Quantikine ELISA: Minneapolis, MN).

Cytokine measurements occurred with the fasting specimen on day 1, and the final postprandial specimens after dinner on days 1 and 2. FFA levels were measured by an enzymatic, colorimetric assay nonesterified fatty acid kit (Wako, Osaka, Japan). This study was approved by the University of Pennsylvania-Institutional Review Board. Written informed consent was obtained from all participants before the implementation of any study procedures and all ethical considerations were incorporated into the IRB review.

### Statistical analysis methods

The random order crossover design was used to reduce potential bias. A sample size of *n* = 16 was determined to achieve 80% power to detect a moderate effect size (*d* = 0.74) to be statistically significant utilizing a one-sample, two-sided *t*-test at alpha level of 0.05% for the primary aim of glucose AUC [PASS 2022 Power Analysis and Sample Size Software (2022). NCSS, LLC. Kaysville, UT; ncss.com/software/pass]. For glucose, FFA, insulin, and cortisol, incremental AUC was calculated using the trapezoidal method for each patient across the measurements obtained at each breakfast, lunch, and dinner on each day 1 and 2. Incremental AUC was used because this was deemed more appropriate in relationship to meals. For each biomarker, AUC was compared between treatments using either the Student's paired *t*-test and/or Wilcoxon signed-rank test, as indicated.

Additional analysis was conducted to assess whether mean biomarker values differed significantly between treatments at specific time points. The effect of treatment order was also evaluated using linear mixed effects models, which were adjusted for treatment order and accounted for correlation among subjects' repeated measures using an unstructured covariance matrix. Cytokine levels were compared between treatments for baseline fasting samples on admission and postdinner samples each day. The statistical level of significance was set at 0.05. Analysis was conducted using SAS 9.4 (SAS, Cary, NC).

## Results

### Demographics

Twelve participants completed the two study admissions and were included in analysis. They represented a racially and ethnically diverse group, including seven Black women, three non-Hispanic White women, one Asian woman, and one Hispanic woman. Demographic results are portrayed as mean values ± SD. The mean age was 33.9 ± 5 years, prepregnancy body mass index = 28.7 ± 5.3 kg/m^2^. The proportions of normal weight, overweight, and obese women were 33%, 17%, and 50%, respectively. Mean gestational age at entrance into study was 32 ± 1.76 weeks, with mean weight of participants at entrance into study (Admission One) = 86.38 ± 16.7 kg and at time of Admission Two = 86.5 ± 16.8 kg.

### Glucose results

**FIG. 3.**
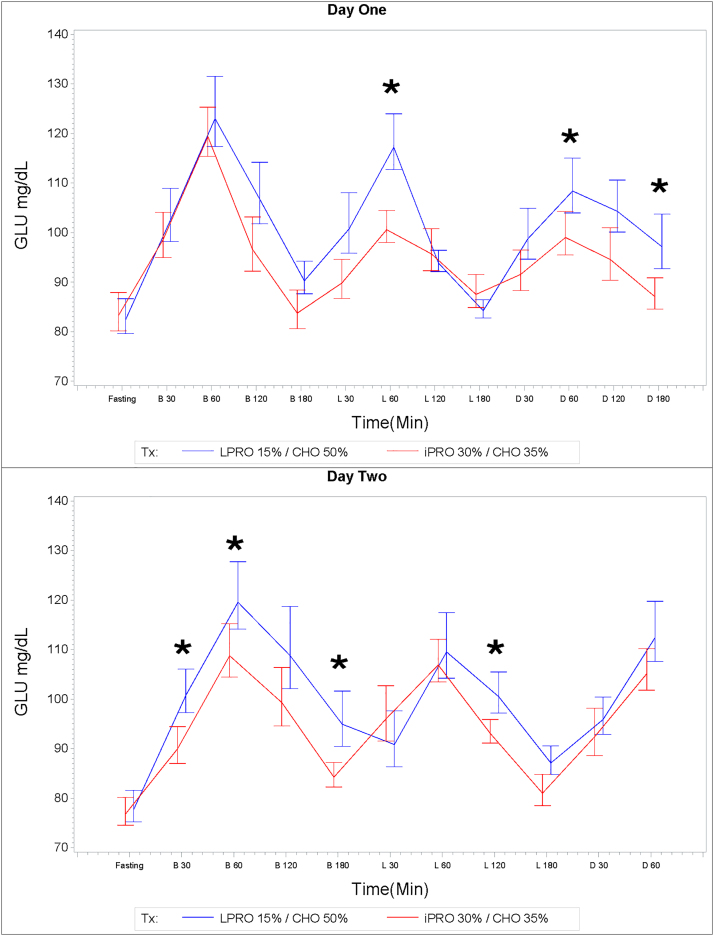
Glucose (mg/dL). Data are mean ± SEM. Asterisks indicate significant difference of mean glucose values at postprandial time points indicated.

On day 2 after an 8-hour fast, the 3-hour postbreakfast glucose AUC was lower in the iPRO30%/CHO35% treatment arm (17395.20 ± 719.80 mg*min/dL) compared to the LPRO15%/CHO50% treatment arm (19172.47 ± 1005.83 mg*min/dL), Student's *t*-test, *p* = 0.01. In addition, in evaluating mean postprandial glucose values over time, the iPRO30%/CHO35% treatment was associated with lower glucose levels with several postprandial measurements when compared with LPRO15%/CHO50% (Fig. 3).

There were three time points after breakfast (30, 60, and 180 minutes) on day 2, where the mean glucose level (mg/dL) was significantly lower with the iPRO30%/CHO35% treatment (90.72 ± 3.72 vs. 101.65 ± 4.38, *p* = 0.03 at 30 minutes; 109.82 ± 5.36 vs. 120.90 ± 6.80, *p* = 0.03 at 60 minutes; and 84.75 ± 2.50 vs. 96.05 ± 5.58, *p* = 0.03 at 180 minutes). Furthermore, there were also significantly lower mean glucose levels with the iPRO30%/CHO35% treatment at 120 minutes postlunch on day 2 (93.51 ± 2.38 vs. 101.33 ± 4.15, *p* = 0.01).

### Insulin results

**FIG. 4.**
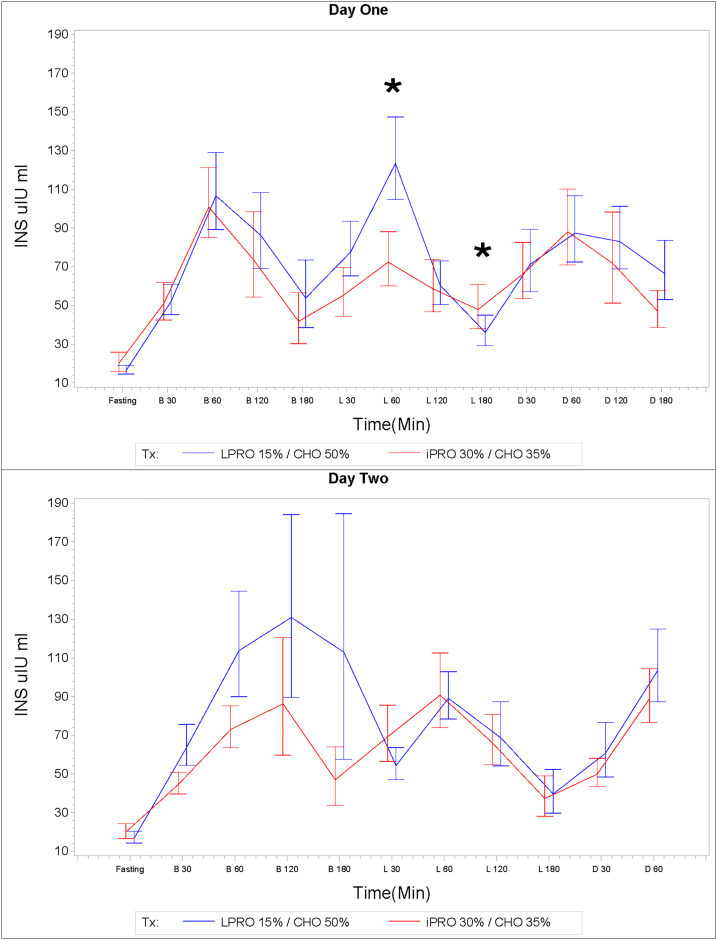
Insulin (μIU/mL) Data are mean ± SEM. Asterisks indicate significant difference of mean insulin values at postprandial time points indicated.

On day 1, the mean 3-hour postlunch insulin AUC was lower in the iPRO30%/CHO35% treatment arm (9281.25 ± 1851.12 μIU*min/mL) compared to the LPRO15%/CHO50% treatment arm (11676.63 ± 1908.46 μIU*min/mL), *p* = 0.0055. One hour after lunch on day 1, the mean insulin value was significantly lower in the iPRO30%/CHO35% treatment arm (74.11 ± 14.02 μIU/mL) compared to the LPRO15%/CHO50% treatment arm (126.08 ± 21.31 μIU/mL), *p* = 0.0034 (Fig. 4). There were no other individual time points where mean insulin values were significantly different.

### Free fatty acids

Both treatment groups exhibited similar trends in FFA millimole (mM/L) levels over the course of the day. The iPRO30%/CHO35% treatment was associated with nonsignificantly higher levels of FFA at most times of the day when compared with the LPRO15%/CHO50%.

**FIG. 5.**
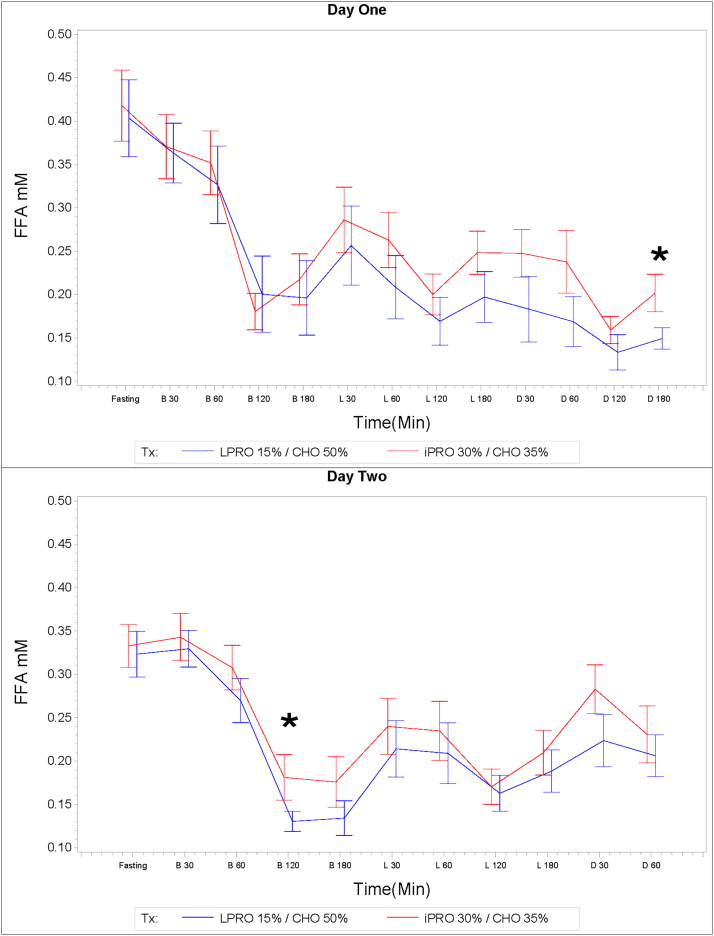
Free fatty acids (mM/L) Data are mean ± SEM. Asterisks indicate significant difference of mean free fatty acid values at postprandial time points indicated.

On day 2, the mean 3-hour postbreakfast FFA AUC was higher in the iPRO30%/CHO35% treatment arm (45.27 ± 3.48 mM*min/L) compared to the LPRO15%/CHO50% treatment arm (38.72 ± 1.87 mM*min/L), *p* = 0.015. There were also two specific time points where there were significant differences. Three hours after dinner on day 1, the mean FFA value was significantly higher in the iPRO30%/CHO35% treatment arm (0.20 + 0.02 mM/L) compared to the LPRO15%/CHO50% treatment arm (0.15 + 0.01 mM/L), *p* = 0.031. Two hours after breakfast on day 2, the mean FFA value was significantly higher in the iPRO30%/CHO35% treatment arm (0.18 + 0.03 mM/L) compared to the LPRO15%/CHO50% treatment arm (0.15 + 0.01 mM/L), *p* = 0.03 (Fig. 5).

### Cortisol

**FIG. 6.**
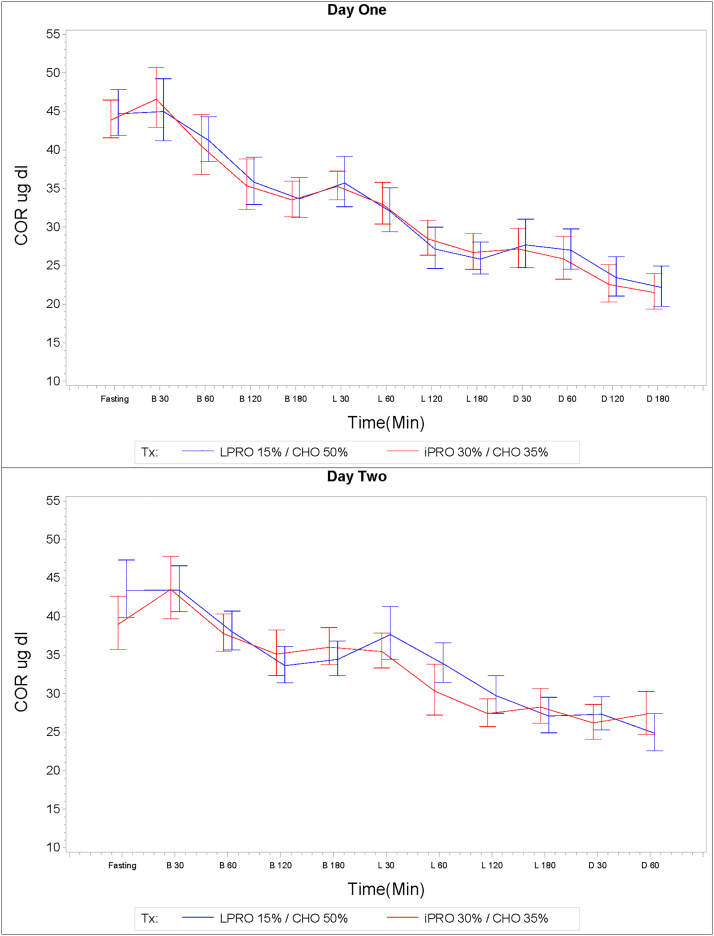
Cortisol: (μg/dL) Data are mean ± SEM. There were no statistically significant differences between the two treatment arms.

Cortisol concentration did not differ by treatment, but followed typical circadian patterns over both days, with mean cortisol levels (μg/dL) higher in the morning with a gradual, continuous decline (except for an increase at 30" postlunch and dinner on both days). Results ranged from a maximum of 67.2 μg/dL in the early morning down to a nadir of 5.5 μg/dL in the evening. Postprandial cortisol concentration AUC was not significantly associated with treatment arm following any meal (*p* > 0.2139). Furthermore, cortisol concentration did not significantly differ by treatment at any time point (*p* > 0.0721) (Fig. 6).

For all analyses described above, analyses adjusted for treatment order yielded consistent findings.

### Cytokines

Levels of proinflammatory cytokines revealed a wide variation between participants at baseline. Concentrations of proinflammatory cytokines (TNF-α, IL-6 or CRP) did not differ between diet treatment arms (data not shown).

## Discussion

This study may be the first to demonstrate that a higher protein and lower carbohydrate meal in women with gestational diabetes can result in lower glucose and insulin AUC and lower postprandial blood glucose values, consistent with our hypothesis. The effect of the iPRO30%/CHO35% treatment, compared with LPRO15%/CHO50%, is particularly evident in terms of postprandial glucose levels following breakfast. Specifically, on day 2, when participants were subject to supervised fasting before breakfast, mean blood glucose levels were significantly lower for the iPRO30%/CHO35% treatment arm at three of the four postbreakfast time points. These differences up to 180 minutes after meals were associated with minimal change in insulin, FFA, cortisol, and proinflammatory cytokines.

Our findings are similar to recent dietary studies in patients with other types of diabetes. The postprandial blood glucose results are consistent with those of a study published by Chang, Francois, & Little who found similar results in a study population of adults with type 2 diabetes.^28^ In another study of adults with type 1 diabetes, a crossover design study revealed that a 40% protein/20% carbohydrate diet resulted in lower glycemic variability and shorter times in hypoglycemia than both a 50% carbohydrate and a Mediterranean diet with 40% carbohydrate.^29^

The ADA consensus report, “Nutrition Therapy for Adults with Diabetes or Prediabetes” (2019) provides recommendations for eating patterns that were supported by research evidence to improve overall health, but does not specify advice for women with GDM.^30^ While there are differing etiologies for the different types of diabetes, one factor that is common to all forms of diabetes is the need to ingest food to survive and the subsequent need to regulate blood glucose. A concern for women with diabetes in pregnancy is potential exposure of the fetus to large amounts of glucose or ketone bodies, with the proper dietary balance helping to avoid either of those extremes. While the evidence for the adverse effects of ketones on fetal and childhood outcomes is conflicting, current clinical guidance suggests that maternal ketosis is to be avoided until further studies can better determine the effects of ketosis on the developing fetus.^31^

Thus, while carbohydrate restriction continues to be advised with GDM, the restriction should not be so extreme as to potentiate maternal ketosis, and in fact, one study found that complex carbohydrate levels could be as high as 60%, yet, still achieve blood glucose targets in women with GDM.^32^ It is not clear what macronutrient proportions are ideal, and they may, in fact, differ for individual patients.

Consistent with the findings of Entringer et al. cortisol levels were highest in the early morning hours for women in the third trimester of pregnancy and constitute an important component of the “awakening response.”^33^ Cortisol is well known to contribute to insulin resistance, and in pregnant women, administration of exogenous corticosteroids to accelerate fetal lung maturity is known to result in a sharp, temporary decline in insulin sensitivity postinjection. Thus, it is not unreasonable to suggest that the endogenous elevations in cortisol found in the early morning hours may contribute to the need to moderate carbohydrate consumption more closely with the early morning meal (breakfast).

A clear strength of this study was the controlled environment in which it occurred, with consistent protocols and precise measurement of all food and beverages consumed. Free-living participants often are unable to adhere to nutrition prescriptions for diet, as other factors such as availability and food insecurity can affect nutritional intake. Our study results may not be replicable when not in a controlled environment.^34–36^ The controlled environment of the research unit may not necessarily translate well into the home environment of the real world of the patient. The influence of increasing placental hormones as advanced gestational age (in the intervening days between the two admissions) could have contributed to a lack of differences between treatments. Other limitations to this study include the small sample size, which may have contributed to type 2 statistical errors in some of the measures.

As we changed both the proportion of protein and carbohydrate concurrently, we are not able to distinguish which component had the greatest impact on the findings. Because of the short duration of our protocol, we cannot project the impact of such an intervention over longer periods of time. While we had no serious adverse events during the feeding trial, we did not monitor the production of ketone bodies. Further, the impact of the higher protein, lower carbohydrate diet on overall health, or safety throughout an entire pregnancy cannot be assumed. While the infants of patients with diabetes infrequently experience fetal growth restriction (unless there is underlying maternal vasculopathy), further study is needed to determine long-term growth implications of increasing protein in the diet.^37^

### Significance

The confusion regarding the most optimal dietary approach for women with GDM can only be resolved by well-controlled studies such as this one. Because we tested glucose, insulin, FFA, and cortisol levels at all time points typically used in the management of these cases, we may be able to describe a protocol that can be used in clinical settings to assess dietary adherence or individual response to diet therapy. Future studies investigating whether broad differences between peak and nadir blood glucose levels (glucose excursions) may contribute to complications of diabetes are needed. The effects of the gut microbiome on glucose metabolism is another area of inquiry demanding further study. There is also no doubt that longer term outcomes of a higher protein diet, including maternal glycemic control, nitrogen balance, and fetal/child growth parameters, are needed.

This study aimed to provide novel data testing higher protein and lower carbohydrate intake as a strategy to achieve improved glucose control in women with GDM. Our precise delivery of dietary treatments in the controlled setting of the clinical translational research center for 36 hours followed by postmeal measurement of insulin and glucose responses is an innovative approach that provided data points with potential for translation into clinical practice settings. Furthermore, because the women who participated were largely from racial/ethnic minority groups with even greater risk of GDM and far less data, the findings are particularly important.

## Abbreviations Used

ADA: American Diabetes Association
AUC: area under the curve
BMI: body mass index
CHPS: Center for Human Phenomic Science
DASH: Dietary Approaches to Stop Hypertension
FFA: free fatty acids
GDM: gestational diabetes mellitus
GI: glycemic index
UPHS: University of Pennsylvania Health System
